# Obinutuzumab-induced coagulopathy in chronic lymphocytic leukaemia with trisomy 12

**DOI:** 10.1038/bcj.2016.42

**Published:** 2016-06-17

**Authors:** H S Walter, S Jayne, P Mensah, F M Miall, M Lyttelton, M J S Dyer

**Affiliations:** 1 Ernest and Helen Scott Haematological Research Institute, University of Leicester, Leicester, UK; 2 Department of Haematology, University Hospitals Leicester, Leicester, UK; 3 Department of Haematology, Kettering Hospital NHS Trust, Kettering, UK

Obinutuzumab, an afucosylated, type 2 anti-CD20 antibody, showed superior results to rituximab in a head-to-head comparison in combination with chlorambucil in the first-line treatment of chronic lymphocytic leukaemia (CLL) patients with comorbidities in the CLL11 trial (NCT01010061).^1^ Enhanced activity of obinutuzumab in this setting may reflect better binding to Fc receptors as well as the direct activation of the lysosomal cell death pathway mediated by type 2 CD20 antibodies.^2, 3^

Nevertheless, obinutuzumab is associated with increased toxicities, mainly infusion-related reactions (IRRs), which occur predominantly during the first antibody infusion, often after only small amounts of antibody.^4^ The frequency of IRR of any grade observed in the CLL11 study was 66% in the obinutuzumab and chlorambucil arm, with 20% being Grade 3–4.^1^ In comparison, only 3% of patients receiving rituximab developed Grade 3–4 IRR. Overall, 7% of patients in CLL11 discontinued obinutuzumab due to severe IRRs.

The precise pathogenic mechanisms underlying these IRRs are not clear. Two recent reports from Freeman *et al.*^4, 5^ have characterised obinutuzumab-associated IRRs in CLL patients. In the first, they demonstrated that IRRs were associated with acute release of cytokines including interleukin 6 (IL6), tumour necrosis factor alpha (TNFA) and interleukin 8 (IL8).^4^ In the second, they correlated levels of CD20 expression with the development of IRRs, higher levels being associated with worse reactions.^5^ Of particular interest was that trisomy 12 was associated with a statistically significant increased risk of IRR; CLL with trisomy 12 exhibit higher levels of expression of CD20. In contrast, *NOTCH1* mutations in CLL (also associated with trisomy 12) are associated with low surface expression of CD20, a feature that may confer poor response to rituximab-based immuno-chemotherapy.^6^

We report a case of CLL exhibiting both trisomy 12 and *NOTCH1* mutation, where an initial 100 mg dose of obinutuzumab resulted in rapid onset of a rare IRR characterised not only by acute severe thrombocytopenia but also disseminated intravascular coagulopathy (DIC).

A 68-year-old female presented in September 2014 with abdominal pain and cervical lymphadenopathy. Blood tests revealed an isolated lymphocytosis (9.9 × 10^9^/l, upper limit of normal 4.0 × 10^9^/l), consistent with CLL on flow cytometry. Computed tomography imaging showed extensive lymphadenopathy above and below the diaphragm, with large-volume (>5 cm diameter) mesenteric lymph nodal involvement. Interphase fluorescent *in situ* hybridisation showed trisomy 12 (55% of cells) and *NOTCH1* mutation, resulting in truncation of the PEST domain, was confirmed by direct Sanger sequencing (Figure 1a). Immunoglobulin heavy-chain variable (*IGHV*) DNA sequence analysis showed 100% homology to *IGHV*1-69; *TP53* was unmutated. Treatment was not required at presentation. One year later she developed symptomatic progressive disease, and in September 2015 she initiated treatment with obinutuzumab as part of an ongoing clinical trial assessing the combination of obinutuzumab and the BCL2 inhibitor, venetoclax (NCT01685892). World Health Organisation (WHO) performance status was 1 and there were no comorbidities. Her white blood cell count was 38.3 × 10^9^/l. Screening coagulation, renal (creatinine clearance >60 ml/min) and hepatic function were normal. She was assigned to start treatment with obinutuzumab before venetoclax. Before receiving the first dose of obinutuzumab (100 mg), she was pre-medicated with 80 mg IV methylprednisolone and 10 mg IV chlorpheniramine. Thirty minutes into the infusion, having received 12.5 mg antibody, she experienced an IRR comprising fever, vomiting, diarrhoea, tachycardia and hypotension. The infusion was stopped and a further dose of IV 80 mg methylprednisolone administered. On resolution of her symptoms, the infusion was restarted and completed as *per* schedule. Abnormal blood parameters were observed on completion of the infusion with the development of grade 3 thrombocytopenia, increase in liver transaminases (grade 1) and rise in lactate dehydrogenase. Coagulation results showed a prolonged prothrombin time of 18.4 s (normal range 12.0–15.0 s), activated partial thromboplastin time of 36.5 s (normal range 24.0–31.0 s), low fibrinogen level of 1.5 g/l (normal range 2.0–4.0 g/l) and D-dimer of >20.00 μg/ml (normal range 0.0–0.5 μg/ml). An acute consumptive coagulopathy was suspected, with subsequent clotting factor assays confirming reduced levels of the other common pathway factors; Factor II 46% (normal range 78.7–115.5), Factor V 47% (normal range 53.8–127.7) and Factor X 64% (normal range 73.1–132.7) (Figures 1b–e). Due to minor epistaxis and low platelets, she received one unit of platelets. Thirty-six hours post-obinutuzumab infusion, the lymphocyte count was only 0.72 × 10^9^/l and palpable lymphadenopathy had resolved. Coagulation factors had normalised aside from a persistent highly elevated D-dimer level, which persisted for the next 3 months. Thrombocytopenia resolved to grade 1 a week later. There was neither clinical nor laboratory evidence of tumour lysis syndrome. Owing to toxicity, treatment was deferred for 3 weeks. Further administration of obinutuzumab proceeded uneventfully. The patient subsequently attained an MRD-negative response in blood and bone marrow (MJSD and HSW unpublished observations).

According to the latest obinutuzumab investigator's brochure (IB September 2015), there have been no previous reports of coagulopathy associated with obinutuzumab and it would appear that this case is a rare event. DIC is a similarly rare complication of rituximab; there have been four reported cases of DIC associated with rituximab infusion (see Thachil *et al.*^7^ and references therein). Interestingly, these cases comprised two cases of Waldenström's macroglobulinaemia and two of hairy cell leukaemia; in only one case did DIC follow single agent rituximab, the rest occurring in combination with chemotherapy. In contrast to acute severe thrombocytopenia, rituximab-induced DIC does not appear to be associated with overt cytokine release syndrome.

The causes of such rare coagulopathies associated with CD20 antibody administration remain unclear but may reflect systemic activation of tissue factor as a consequence of rapid antibody-mediated tumour cell depletion. It should be noted in this case, however, that despite the very rapid clearance from the peripheral blood and lymph nodes, there was no evidence of tumour lysis syndrome. Tumour lysis syndrome has been seen in CLL treated with single agent obinutuzumab,^8^ but may be more common when given in combination with bendamustine (Addendum to obinutuzumab IB, March 2016). Interestingly, this very rapid tumour reduction occurred in the presence of *NOTCH1* mutation; whether obinutuzumab can overcome the negative impact of this mutation in CLL awaits further studies.

## Figures and Tables

**Figure 1:**
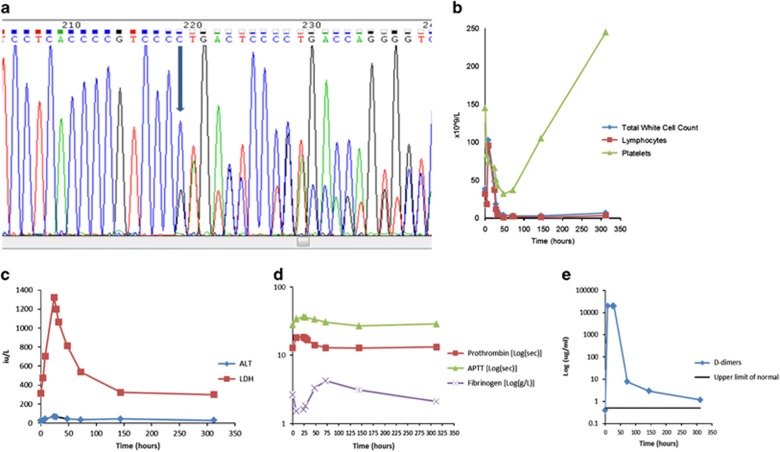
(**a**) Sequencing trace showing the NOTCH1 mutation c.7544_7545delCT mutation (RefSeq NM_017617.2); arrows point to the position of the nucleotide change. (**b**–**e**) show abnormal blood parameters observed on completion of the infusion.

## References

[bib1] Goede V, Fischer K, Busch R, Engelke A, Eichhorst B, Wendtner CM et al. Obinutuzumab plus chlorambucil in patients with CLL and coexisting conditions. N Engl J Med 2014; 370: 1101–1110. 24401022 10.1056/NEJMoa1313984

[bib2] Mossner E, Brunker P, Moser S, Puntener U, Schmidt C, Herter S et al. Increasing the efficacy of CD20 antibody therapy through the engineering of a new type II anti-CD20 antibody with enhanced direct and immune effector cell-mediated B-cell cytotoxicity. Blood 2010; 115: 4393–4402. 20194898 10.1182/blood-2009-06-225979 PMC2881503

[bib3] Alduaij W, Ivanov A, Honeychurch J, Cheadle EJ, Potluri S, Lim SH et al. Novel type II anti-CD20 monoclonal antibody (GA101) evokes homotypic adhesion and actin-dependent, lysosome-mediated cell death in B-cell malignancies. Blood 2011; 117: 4519–4529. 21378274 10.1182/blood-2010-07-296913 PMC3099571

[bib4] Freeman CL, Morschhauser F, Sehn L, Dixon M, Houghton R, Lamy T et al. Cytokine release in patients with CLL treated with obinutuzumab and possible relationship with infusion-related reactions. Blood 2015; 126: 2646–2649. 26447188 10.1182/blood-2015-09-670802 PMC4671111

[bib5] Freeman CL, Dixon M, Houghton R, Kreuzer KA, Fingerle-Rowson G, Herling M et al. Role of CD20 expression and other pre-treatment risk factors in the development of infusion-related reactions in patients with CLL treated with obinutuzumab. Leukemia 2016, ; e-pub ahead of print 8 April 2016 doi:10.1038/leu.2016.41. 10.1038/leu.2016.41 PMC4980557 26979130

[bib6] Pozzo F, Bittolo T, Arruga F, Bulian P, Macor P, Tissino E et al. NOTCH1 mutations associate with low CD20 level in chronic lymphocytic leukemia: evidence for a NOTCH1 mutation-driven epigenetic dysregulation. Leukemia 2016; 30: 182–189. 26165233 10.1038/leu.2015.182

[bib7] Thachil J, Mukherje K, Woodcock B. Rituximab-induced haemorrhagic thrombocytopenia in a patient with hairy cell leukaemia. Br J Haematol 2006; 135: 273–274. 16965386 10.1111/j.1365-2141.2006.06299.x

[bib8] Byrd JC, Flynn JM, Kipps TJ, Boxer M, Kolibaba KS, Carlile DJ et al. Randomized phase 2 study of obinutuzumab monotherapy in symptomatic, previously untreated chronic lymphocytic leukemia. Blood 2016; 127: 79–86. 26472752 10.1182/blood-2015-03-634394 PMC4705612

